# Perspectives on healthcare artificial intelligence policy from health equity professionals: findings from an interview study

**DOI:** 10.3389/fdgth.2026.1797705

**Published:** 2026-05-07

**Authors:** Kadija Ferryman, Odia Kane

**Affiliations:** 1 Johns Hopkins Berman Institute of Bioethics, Baltimore, MD, United States; 2 Department of Health Policy and Management, Johns Hopkins Bloomberg School of Public Health, Baltimore, MD, United States

**Keywords:** aritifical intelligence, community experts, digital health, health AI, health equity, health policy

## Abstract

**Introduction:**

While artificial intelligence tools (AI) can have positive impacts on healthcare, such as by expediting administrative tasks and aiding in clinical decision-making, recent studies have shown that AI-enabled systems can perpetuate biases against medically underserved populations which can exacerbate health disparities.

**Methods:**

This qualitative interview study solicited the perspectives of professionals who work on issues related to health disparities and health equity to understand how health AI policy could be developed with considerations of addressing these issues. We conducted semi-structured interviews with individuals (*n* = 22) who work at organizations or serve in roles that focus on health disparities, promote health equity, and or primarily support health for underserved populations across the United States. The interview guide was developed using Michener's Racial Equity Framework for Assessing Health Policy with questions that focused on actors, networks, institutions, and contexts that are relevant to artificial intelligence, health equity, and health policy.

**Results:**

Three key themes emerged from our thematic analysis: 1) developing health AI policy for health equity starts with data, 2) health AI policy for health equity must include multiple institutions and strategies, and 3) considering economic issues is key for developing health AI policy that advances health equity. Participants highlighted the importance of interdisciplinary cross-sectoral collaboration to address the structural issues that contribute to health disparities as important for developing policy for health AI tools.

**Discussion:**

The findings of this study show that health equity professionals have specialized knowledge and offer unique insights important to emerging policy areas for health AI. This is the first study to focus on the views of this specific group on the development of health AI policy for health equity.

## Introduction

Artificial intelligence (AI) tools can be used in health care in numerous ways, including for administrative tasks like appointment scheduling to direct clinical functions like identifying cancerous tumors in medical images (1, 2). Policy guidelines and governance of these tools in healthcare are in nascent stages. At the federal level, there are definitions of what constitutes AI as a medical device, but which kinds of AI tools are subject to regulation by the Food and Drug Administration (FDA) has been the subject of recent clarification due to noted problems with AI used in hospitals which were not FDA-regulated (3). In addition, despite the recent passage of Section 1557 of the Affordable Care Act which outlaws discrimination by clinical algorithms, attention to how to craft and align health AI policy while mitigating health disparities *and* advancing health equity has been limited at various levels of governance (4).

There is a growing body of scholarship on digital inclusion in health and digital health equity that argues for the development of technology which can help mitigate health disparities and offers guidance on how to engage marginalized groups (5–7). There is also literature more pointedly focused on healthcare artificial intelligence and health equity, which largely focuses on how to mitigate bias in AI in order to mitigate health disparities and advance health equity and how to engage community and marginalized groups (8–12). This work provides important conceptual frameworks and guidance for AI development and implementation such as Chen and colleagues' justice-informed framework for AI development and the Health Equity Across the AI Lifecycle (HEAAL) framework (13, 14).

Although this work surfaces important issues of AI bias and provides guidance for ways to address and mitigate it, this work is largely conceptual. There is some notable empirical-based work which focuses on healthcare AI and health equity, but there is less specific engagement with how health equity and AI issues are relevant for policy, especially for policy at multiple levels, from institutional to federal policy (15–17).

This gap in empirically-grounded research at the nexus of healthcare AI, health disparities and equity, and multi-level policy is important to address. To that end, we undertook research guided by the question: How might input from those who focus on health disparities and/or racial and ethnic minority health affect policy development and governance for health AI? We argue that it is imperative to gather information from those working to address health inequities to understand how they view health AI regulation and how they might contribute to policy development in this area.

## Materials and methods

In this study, we focus specifically on professionals in the health equity domain. This is because we identified a gap in the literature at the intersection of health AI and health equity. While there is literature that includes patients' views of health equity in digital health and health AI, there is little work which focuses specifically on the perspectives of those who have expertise in this domain (15). In addition, although there is research showing that health disparities researchers are disfavored for National Institutes of Health (NIH) grants (18) and that they face marginalization as professionals (19), we found no research centering the viewpoints of health disparities professionals on health equity in AI. Our intention was to focus on professionals whose work primarily focuses on health equity in order to examine their views on challenges and facilitators to AI health policy development. We define our respondents as health equity professionals because they work at minority health organizations such as the National Hispanic Health Foundation. We define “minority health organizations” broadly as organizations doing research, advocacy, policy development, or other activities with a focus on minority health in the US. We also considered health equity professionals to be people who may not be working at a minority health organization but their professional work activities (which could be advocacy, research, or other kind of work) focuses on minority health disparities and/or health equity.

We conducted interviews with individuals who work at organizations that focus on health disparities and/or health equity, individuals at organizations that primarily serve marginalized populations, and individuals who focus on health disparities within larger organizations (*N* = 22). Drawing on professional networks, snowball sampling, online searches for professionals at minority health organizations and within organizations with a focus on health disparities, we used a purposive strategy to recruit interview participants. Participants were sent recruitment emails with information about the study and interviews were scheduled via email. One author (KF) developed a semi-structured interview guide drawing from Michener's Racial Equity Framework for Assessing Health Policy (20). Interview questions focused on actors, networks, institutions, and contexts that are relevant to artificial intelligence, health equity, and health policy. This interview guide was reviewed by five doctoral-level research experts, including four at Johns Hopkins and one who is external and was then refined by the study authors (supplementary S1 Text). We did not collect demographic data from respondents, such as age, race/ethnicity, and gender for two main reasons. First, because of the relatively small study sample and the small population of health equity professionals who are also well versed in health AI and digital health, we decided not to collect demographic information to reduce re-identification risks. In other words, we did not collect this information to preserve the privacy of our interview respondents. The second reason is methodological feasibility. Because this is a qualitative study with a small sample, analyzing responses by demographic characteristics to calculate association between demographic characteristics and themes would not be possible or desirable. The Johns Hopkins Institutional Review Board (IRB) reviewed the study and it was determined to be exempt. Oral consent for participants was permitted by the IRB.

Semi-structured interviews were conducted via videoconference by both authors and were recorded and transcribed. Interviews ranged from 36 min to 65 min. Key points and issues raised in the interviews were discussed by the authors after each interview. Interviews were concluded when the authors determined, through discussion, that no significant new information was raised in the interviews. Both authors hand-coded the same interview transcript separately to identify codes from the text. These initial codes were discussed, combined, and refined into an initial codebook (Supplementary S2 Table). This initial codebook was used to code a second transcript by both authors using the qualitative analysis software Atlas.ti. The authors then discussed the results of coding the second transcript to further refine the initial codebook into a final codebook. The authors then split up the remaining transcripts and coded them separately using the final codebook. After the transcripts were coded, the authors reviewed each other's coded transcripts to check for any deviations and to discuss discrepancies. The coded excerpts were then read and analyzed inductively for themes by both authors. Our conception of a theme is related to Braun and Clarke's description of a theme as a pattern of shared meaning (21). Each author read the coded excerpts to identify clusters or “buckets” of similar meaning both within and across the coded excerpts and created memos that described patterns of shared meaning across the excerpts. The authors read each other's memos and discussed the identified patterns. Shared patterns of meanings were condensed into themes, and thematic saturation was reached when no new themes were identified by the authors. The authors created thematic memos which included interview excerpts and these memos were used to write this manuscript.

### Positionality statement

The research team consisted of a faculty researcher trained in anthropology, policy research, and critical data studies (KF) and PhD student (OK) with training in political science, bioethics, and health policy. Both authors have held professional positions in policy research organizations. In addition to educational and professional experiences, both authors are first-generation Americans born to immigrant parents from the Global South. Both authors also have lived experiences with marginalized and intersectional identities, which undergird our interest in the topic of health equity, but also shapes our understanding of the topic. We recognize that our educational, professional, and personal experiences shape our approach to the research and our interpretation of the results (22).

## Results

Our respondents work in different kinds of organizations and in various roles whichinclude patient advocates who have founded organizations dedicated to closing health equity gaps, community activists, health equity-focused academics, scientists, community-based physicians as well as those with technical skills and understanding of AI and software development. More specifically, our participants fell into four categories, 1) those who worked at a minority health organization, 2) those who were at academic institutions conducting research with a focus on health disparities or health equity 3) those who worked at a health care provider organization with a focus on health disparities or health equity, and 4) one who worked in private industry with a focus on health equity (Table 1). In terms of population focus, most of our study participants focused on a specific minoritized group in their research and/or advocacy (Table 2). For example, respondents who worked at a specific minority health organization, also focused on that specific population in their work. Although we did not intend for our respondents to be nationally representative, the majority of our participants worked in the southern and western regions of the United States (Table 3). We did not ask participants to provide any personal demographic information about themselves, such as their age or gender.

**Table 1:** Participants' Organizations.

Participants' Organizations	Count
Minority health organization	6
Academic center, department, or in position that focuses on health disparities or health equity	10
Health care provider organization with a focus on health disparities or health equity	5
Private industry with a focus on health equity	1
Total	22

**Table 2:** Participants' population focus.

Participants' Population Focus	
Their work focuses on a specific minoritized group or population	12
Their work does not have a specific minoritized group or population focus, but has a broad focus on health disparities	10
Total	22

**Table 3:** Geographic distribution of interview participants.

Geographic Distribution of Participants	
Northeast	3
Midwest	2
South	10
West	7
Total	22

Participants discussed health AI, health disparities, health equity, and policy development in three key themes: 1) developing health AI policy for health equity starts with data 2) health AI policy for health equity must include multiple institutions and strategies and 3) considering economic issues is key for developing health AI policy that advances health equity (Table 5).

### Theme 1: developing health AI policy for health equity starts with data

Participants mentioned that health policy for AI which mitigates health disparities and advances health equity begins with data, specifically identifying what its problems are, and how to get better data.

#### Problems with data that can be used to train health AI

Several participants noted that health AI should be trained on diverse data, so that AI tools do not end up either exacerbating existing health disparities or creating new ones. Participants noted that diversity can be lacking in health data, such as in genomic databases. Even though there has been recent attention to diversifying these databases, they are still over-represented by individuals with European ancestry (23). A respondent [10015] also cited lack of database diversity in the International Skin Imaging Collaboration database. This is important because this database can be used to build AI tools that detect skin conditions, but the lack of diversity can mean that these tools could work less well on those who are not adequately represented in the dataset (see illustrative quote in Table 4). This lack of diversity in datasets can be so dangerous, that the participant suggested that databases should have advisory labels like records do, warning potential users of what they do–and do not–contain in terms of population representation.

**Table 4:** Illustrative quotes.

Respondent ID	Theme	Illustrative Quote
100,105	Developing health AI policy for health equity starts with data	“… the International Skin Imaging Collaboration has tens of thousands, I think, like [16000] images that chronicle different forms of popular skin issues that affect the patients that have submitted the data. But the problem is that those patient pools come from–they're all white. Basically, they're like ninety-nine percent white, all those photos in there, like ninety-nine percent white. They source the data in Europe or Western Europe, United States and Australia.”
10,001	Health AI policy for health equity must include multiple institutions and strategies	“when we see actual algorithms that can have an impact versus, “Oh, here's a new tool that could hypothetically, potentially have an impact where I used a toy data set” … it is tailored for that problem, having deeply understood that problem. And then, leveraging those relationships with policymakers to see, “Okay, how could we potentially embed this in [the] future?” … building in fairness considerations tailored towards solving a health equity problem has definitely shown that there's a potential path forward for these tools …”
10,009	Considering economic issues is key for developing health AI policy that advances health equity	“we say, let's look at the ten percent of health plan patients that are attributed to us that have the most medical complications–high-risk, high-cost per capita, and let's co-manage that risk pool and let's invest in the community to address social needs … we work with the health plan and mutually agree on a cohort of about ten percent of the patients they attribute to us and we are capitated or incentivized to provide care coordination. And we run the [AI] tool to measure the impact of social interventions along with clinical interventions, and what works to improve the quality outcomes and cost savings.”

**Table 5:** Summary of respondents' recommendations for health equity in AI policy.

Theme	Recommendations
Theme 1: Developing health AI policy for health equity starts with data	Increase community diversity and representation in datasets, add “warning label” to datasets which lack diversity Learn about how social contexts and biases are embedded in health data Identify power asymmetries in data collection and build structures for community access to health data for AI Encourage collection and inclusion of data that gives a fuller picture of health and life history, such as data from caregivers
Theme 2: Health AI policy for health equity must include multiple institutions and strategies	Longer term commitments from funders for AI and health disparities research, also include small research institutes; encourage academic and community research partnerships to investigate health equity in AI Encourage federal agencies to investigate how health AI tools may violate rights Include robust evidence on AI benefit and equity as part of regulating health AI, such as clinical trials as part of FDA AI regulation Encourage accreditors such as Joint Commissions to include health equity in assessment of health AI
Theme 3: Considering economic issues is key for developing health AI policy that advances health equity	Use AI tools to identify high-needs patients without expecting organizations to “do more with less” Pitch equitable AI as a marketing strategy Invest in under-resourced healthcare facilities to increase capacity to use and maintain AI solutions Consider how integration of health AI tools can have workforce implications

Another participant noted that data that come from health systems that are used for AI development can reflect existing health inequities. For example, respondent 10,004 noted that health data comes from the “flawed health system.” One respondent said that social determinants of health are the “context that shapes the data,” while another noted that social determinants of health shape the data, but can also be conceptualized as innumerable “unmeasured variables” (10014) that may not be easily captured with conventional measures. Participants (10011, 10018) noted that AI developers may not know about how social conditions can affect health data, such as how racial segregation is connected to health disparities. This lack of knowledge can contribute to AI tools being biased against minoritized groups. Similarly, another respondent mentioned that societal issues can be embedded in health data, even when not explicit as a variable. One respondent noted that race and racism are present in datasets, even if race is not an explicit variable, calling it a “ghost” and a “shadow” in health data:

“Race will persist as the ghost in the machine … since race correlates with so many other variables in American society … experiences of racism, access to healthcare spending … as the shadow of race as a ghost in these data sets, even if race isn't actually encoded in the dataset” (Respondent 10023).

Several participants (10004, 10010, 10018) mentioned that there is bias and systemic racism embedded in the health care system, which is reflected in health data. One participant, who is a Black woman with a PhD, saw that her health records identified her as having less than a high school diploma. She argued that racist assumptions like these make their way into health data which can then influence AI built using this data.

#### How to get better data

Participants also discussed how power asymmetries relate to data in health AI development, and that is important for considering how to develop health AI policy that addresses health inequities. They noted power differences between laypersons/community members vs. scientists and AI developers, and between AI developers and technology companies vs. health care provider organizations. Respondent 10003 noted that health insurance companies are in a powerful position because they have a lot of data about individuals and are in a position to develop AI tools with this data for their own financial benefit, and not necessarily for patients' benefit. Another respondent mentioned the importance of providing laypersons and community groups access to data that can be used to develop health AI. Respondent 10004 argued that in order to address data asymmetries, there needs to be new data governance structures and “different models of engagement … having these tools means really rethinking a lot of the systems around the tools, a lot of the power differentials [and] whose needs are seen as more important.” Relatedly, one respondent noted that getting health data can be an “extractive” process, and that measures should be taken to ensure that there is mutual benefit:

“communities who want the data, who are generating the data, and that either they collect about themselves or that others collect about them, you have large institutions that, you know, deploy the data collection tools. The large organizations benefit from the use of that data, but again they're larger organizations that have a lot more power in the communities too, so, for one, recognizing that the communities have access to the data that's being collected about them. Access such that they don't need to overcome paywalls or overcome significant technical boundaries that might exist around them accessing data collected about them … Do the pipelines exist for communities to benefit from that data in a way that empowers and enriches? In [a way] that helps communities address, you know, the needs that they have right then and now …  organizations that collect data or extract data from and about communities. What are they responsible for? Who's holding them accountable to the communities? How has the Community been empowered to at least gain some control or power over how their data is being collected and used? How they're being surveilled? Those sorts of things are things that we think about … ways of balancing those power asymmetries … ” (Respondent 10005).

Also, this respondent (10005) noted that there is a tension when thinking about data for health AI–it provides insight, but it is also a valuable “commodity” that can be bought, sold, and kept for private use by powerful institutions who do not want to share their data.

Participants also mentioned several ways to augment data that can be used to train health AI, so that these tools will not exacerbate health inequities. Respondent 10005 noted that one way to address health disparities is to get comprehensive “holistic” data that informs policy or can augment, such as collecting not just patient data but data from patients' caregivers. Respondent 10021 mentioned that collecting patient data over the life course could be another way to capture social determinants of health data and that this might in turn mitigate potential harms of health AI tools, because this data could provide a more comprehensive view of the patient's life history.

The participants' comments about attention needed to the ways health system bias and social discrimination affect health data and that power asymmetries exist in health data access and use is important to consider alongside current efforts in synthetic data generation, specifically how the production of this data can make up for some of the gaps, limitations, and biases in healthcare data (24). Although there is some evidence that generating synthetic data can be one way to achieve increased data diversity, our respondents noted that biases are often embedded within data, so having more representative data on populations may not safeguard against pernicious bias in subsequent health AI tools. In addition, our participants noted that the process of getting better data should involve shifts in power in data collection and use, which means that diverse synthetic data created within existing power structures may only address part of their concern.

### Theme 2: health AI policy for health equity must include multiple institutions and strategies

Respondents discussed that policy for health AI that focuses on health equity must occur at multiple levels of governance and involve multiple and overlapping strategies. Study participants mentioned opportunities for policy development by federal agencies, health systems, nonprofit organizations and academia.

#### Federal level

Respondents identified agencies like the Center for Medicaid and Medicare Services (CMS), the U.S. Food and Drug Administration (FDA), and the National Institutes of Health (NIH) as the main policy actors at the federal level. Respondent 10005 mentioned that major funders like the NIH need to “commit to [the] long term,” because health disparities cannot be addressed by 2-year research projects. Respondent 10,023 mentioned that the Department of Justice is an important institution in AI policy that could center health equity, because it can investigate how the use of medical tools can violate civil rights. Another respondent noted that the FDA could use clinical trials to measure the health equity implications of health AI tools. Respondent 10009 mentioned that CMS could wield its influence as a health care coverage provider to influence health AI developers to focus on equity. CMS has already taken steps in this direction, for example, their Technical Assistance Program supports health care organizations in identifying social needs and leveraging data on health disparities in their patient populations to better embed health equity into organizational strategic plans, and our respondent's suggestion provides a way to build on that effort with a focus on health AI.

#### Health systems, nonprofits, and academia

Respondents mentioned that organizations such as the Joint Commission could leverage their influence on health systems as an accreditor to emphasize health equity in health AI tools. The Joint Commission has an Excellent Health Outcomes for All certification that focuses on health equity as well as responsible use for AI guidance developed in collaboration with the Coalition for Health AI. Our respondents' suggestion points to standard-setting and evaluation organizations like the Joint Commission as an important actor in policy development, since they can influence organizational change and regulation outside of formal government regulation.

Respondents 10011 and 10006 mentioned the importance of developing internal policies within healthcare organizations to help ensure that AI tools are not worsening health disparities. Specifically, respondent 10006 argued that chief executive officers and chief medical officers of major academic medical centers can influence policy relating to health disparities. However, when discussing internal policies in healthcare provider organizations, respondent 10016 mentioned the importance of making sure that policies, which may be made by hospital executives, are legible and implementable for frontline physicians and nurses. In addition, respondent 10001 also highlighted the role that clinicians themselves can play in exacerbating health inequities, for example, by “gatekeeping” and not providing equal or equitable access to health technologies for their patients (see illustrative quote in Table 4).

Several respondents also mentioned that academic organizations can also influence health AI policy to address bias and facilitate health equity, and one important way can be through researchers engaging with patients and community members. Respondents 10006 and 10011 stressed the importance of researchers engaging with the community because AI is “just a tool” and even if it “works,” still needs trust from patients. These respondents noted that patients and community should be both consulted and be active participants in designing research and policy for health AI. Two respondents noted that “small research institutes” can act as “equity partner(s)” when thinking about how to develop health AI tools and governance that promote health equity, not just large academic institutions. These comments dovetail with the growing literature on community engagement in healthcare AI (24). For example, Nong writes that “data use for algorithmic development is becoming part of the trust calculation that patients make as they seek healthcare” (25). Other scholars discuss the importance of involving community members in creating natural language processing tools for use in healthcare (26). Further, the recent code of conduct for healthcare AI developed by the National Academy of Medicine highlights engagement with stakeholders as a key commitment (27).

Some participants expressed skepticism noting possible tensions with cross-sector collaborations, even if facilitated by academia, because they could thwart health equity aspirations. One respondent (10018) mentioned a distrust of corporate influences in government and communities stating “ … So to be honest, I don't know what the difference is any more between corporate and for-profit entities and the government, unless you build a structure that makes them immune to that financial and push that effect of financing on your ability to function, and Congress will approve things that will benefit the people that fund their campaign regardless if it's good or bad. And that's the problem …” Respondent 10023 said that AI tools should be subjected to “much greater scrutiny than we have thus far otherwise we're at risk of getting sent on a detour to some very not ideal future” but had some reservations about whether all relevant stakeholders would commit to creating and adhering to stringent guidelines. Respondent 10009 warned against health systems and private corporations overlooking financial burdens patients can incur through increased costs as a result of investment in AI-enabled services or procedures (e.g., robotic-assisted surgeries) (28).

### Theme 3: considering economic issues is key for developing health AI policy that advances health equity

Several respondents discussed financial and economic aspects of developing health AI policy for health equity. For example, some mentioned developing risk tiers of patients using AI tools (10008, 10019, 10020), and how these risk tiers can help them make better use of fixed payments they receive from health insurance plans. Several respondents specifically discussed how measuring social determinants of health could be used to identify high risk patients using AI, and that this could lead to financial benefits for healthcare provider organizations. For example, respondent 10009 noted that identifying social needs could help healthcare organizations increase their revenue, and that “social needs are the source of a lot of preventable events” (see illustrative quote in Table 4). Although identifying patients at risk using AI could be beneficial to both patients and health care provider organizations, one respondent (10001) mentioned a danger of using Al to identify high-risk patients: this strategy could help hospitals save money, especially those who serve high-needs patients, but it may also enable these same organizations to do more with fewer resources, without addressing root causes of their patients' needs and the institutions' limited resources. For example, respondent 10023 mentioned that the Indian Health Service (IHS) gets less funding than other federal health programs, even though many of the patients they see are “triple eligibles” for Medicare, Medicaid, and IHS funds. Another respondent mentioned that AI could be used to examine disparities in how hospitals are receiving funding (10018), while another (10021) mentioned that AI could be used to optimize grant funding in order to prioritize high-needs patients and health disparities. These efforts, in their view, would be economically-driven health AI policies that could be developed by institutions and at the federal level to help mitigate health disparities.

Respondents also mentioned that highlighting AI tools that are equitable and do not exhibit bias could be a way to make them more commercially successful, or could be a way of “marketing for equity.” This is important especially as venture capital is an important investor in developing health AI (10023). Importantly, one respondent (10002) mentioned the connection between regulation and cost: although more regulation of AI tools, especially for health equity is desirable, they argued that it is also important to consider that more regulation could lead to higher cost of health AI products, as the costs for meeting regulatory guidelines could be passed on to the consumer. More expensive health AI tools, especially those which can meet potential equity guidelines and policies could create another inequity: lower-resourced healthcare provider organizations may be less able to acquire more expensive, though more equitable, health AI, suggesting that health equity centered AI policy would need to balance these tradeoffs. Respondent 10018 mentioned that the cost of health technology like AI is a structural dilemma: hospitals may be able to acquire a technology but struggle with its maintenance costs. At the same time, healthcare provider organizations may also need to purchase cutting-edge technologies to attract more clinicians, patients, and bring in more revenue.

One respondent (10015) mentioned that financial incentives may prevent the development of health AI policy that encourages health equity, because abiding by health equity policy or showing that their tools are mitigating bias may demand opening the “black box” of the AI, and revealing aspects about their technology that they may want to remain out of public view as part of their “competitive advantage”.

#### Skepticism of AI for health equity

Despite respondents discussing opportunities for health AI policy development that emphasizes health equity, some respondents were skeptical about the development of health AI in general, and whether it is “solving real problems” (10017). Respondents mentioned the concern of AI developers may reinforce existing “feedback loops” (10016) in clinical care that overlook disparate outcomes, go on “fishing expeditions” (10021) that propose solutions to insignificant problems for profit, or make design choices that undermine health equity efforts. This sentiment is aligned with studies of healthcare professionals' views of healthcare AI, which raise the concern of the use of patient data by organizations which may have profit generation, rather than patient care, as a primary motivator (29, 30).

One respondent 10014 explained that even when algorithms produce fair and equitable outcomes, dissemination of this information is another challenge. “So far we have a validated stroke identification algorithm … shown to have high accuracy for identifying patients with stroke, no matter their race and ethnic background. And so we've shown that now publishing that has been difficult, and there's a side story. It's sometimes difficult to focus on the health disparities aspect of publications … I've seen all types of reviews that are problematic.” This comment reflects the research which shows that health disparities research and topics are marginalized by biomedical institutions (30). Thus, this respondent's skepticism of the use of AI for health disparities may be rooted in trends that pre-date the widespread use of health AI technologies.

Also, some respondents mentioned that focusing attention on how to make health AI more focused on health equity can detract attention from people who have been doing health equity research for a while, because “Al is flashy.” This relates to the critique of the recent influx of health disparities researchers from historically privileged backgrounds, referred to as “health equity tourism” (31). One respondent did not believe that AI could meaningfully contribute to addressing health inequities (10012) because “there's so many things that happen before you show up to that space.” Respondent 10011 discussed conversations with AI developers who were seemingly unfazed by cascading negative consequences of high investments in AI/ML solutions, like worker displacement. “I had started talking to these young men … and they started talking about how great AI is and I said it's going to put janitors out of work. Have you thought about that yet?  …  [Y]ou know, jobs are dignity. This is not about money it's about dignity and integrity  …  (10011).” The National Academy of Medicine's code of conduct for healthcare AI includes attention to workforce issues as a key commitment for ensuring responsible AI, but some researchers argue that ethical considerations may be an “afterthought” for health AI developers (32).

## Discussion

When asked about important actors, networks, institutions, and contexts for health AI policy development that would address health inequities, respondents who work explicitly on health disparities and health equity provided a range of insights, clustered in discussions on the importance of data, multiple institutions and strategies, and financial considerations that could affect policy development. Respondents discussed how specific kinds of organizations such as health systems and academia can draw on their spheres of influence to contribute to health AI policy development, but they also noted that institutions are enmeshed in networks where there are unequal power relationships. They argued that it is important to acknowledge these differences, but they do not have to stymie the development of health AI policy that can mitigate health disparities and advance health equity.

Many of the respondents drew on their close experience with health disparities–providing care to people subject to health inequities, conducting research on health disparities, and participating in advocacy and action for marginalized groups–to inform their thoughts on how policy for health AI can focus on mitigating health disparities and advancing health equity. The view of health disparities as structural influenced how they discussed health AI policy, such as identifying structural economic issues like differences in hospital funding as an important consideration for health equity policy for AI.

The themes distilled from the interviews have some overlap with but are distinct from published FDA guidance regarding medical device AI. For example, early FDA guidance on medical AI did not mention AI bias, health equity, or health disparities (33). The most recent guidance from the agency does include a focus on AI bias and mentions the importance of data representativeness in AI to help address this issue. This dovetails with participants' comments about the importance of data for AI policy to advance health equity. In addition, this guidance does not mention multi-stakeholder collaboration, nor does it mention how economic issues might affect the testing, use, or post market monitoring of medical AI. This suggests that garnering the perspectives of health equity professionals is important because they bring a distinct set of issues to the fore (12).

Another relevant recent federal health AI policy is Section 1557 of the Affordable Care Act, which outlaws discrimination in healthcare, including by clinical algorithms. Thinking about this rule with our three themes allows for consideration of how data, multiple stakeholders, and economic issues are key considerations for avoiding discrimination in clinical algorithms. The comments from our respondents show that there is complexity to this legislation, which at its face seems like an obvious win for health equity. Our respondents emphasized structural differences like how resources are unevenly distributed and captured by different healthcare institutions in the US, which means that although Section 1557 is intended to advance health equity, it could exacerbate institutional inequities (34). For example, healthcare institutions must invest in AI infrastructure, including computer systems and human expertise to not only implement health AI, but be able to monitor and audit it for potential discriminatory harms. If under-resourced organizations have less capacity to do this, Section 1557 could add another inequity to these organizations. It is also important to note that this policy may not be enforced by the Trump Administration due to significant staff changes at the Department of Health and Human Services, as well as the administration's view that diversity, equity, and inclusion efforts are unlawful (35, 36).

Based on our themes, we recommend that AI developers, health systems, public health practitioners, health equity scholars, and organizations that represent key users such as healthcare providers, patient advocacy groups, and caregiver advocacy groups form a national coalition on health equity standards for AI. There is already some momentum in this area, for example, the Health AI Partnership has produced the Health Equity Across the AI Lifecycle (HEAAL) framework, which aims to help healthcare organizations with decisions surrounding adopting new AI technology (9). The coalition that we suggest here could bring together healthcare organizations who are implementing HEAAL and other health equity efforts in order to share best practices, challenges, and create opportunities for alignment and collaboration. This group could gather resources and recommendations for equitable health AI development and use, including advocating for the broader use and funding of diverse health data, as well as serve as a learning community and community of practice. In line with recommendations from our respondents, this coalition could help foster better community “pipelines” for access to and support in interpreting health data used for health AI (35). The NIH's Bridge2AI program is a promising example of a way to broaden access to health data for AI, and can provide a model for how to “ethically source” health data (37). The coalition could further work to build and refine strategies for communities to use this ethically-sourced data for health AI. The coalition's national influence could be leveraged to concretize these strategies into policy at the institutional, state, and federal levels. The coalition could also discuss marketing health AI for health equity as our respondents suggested. This group could also decide on standards and evidence needed for health AI tools to be certified by them as mitigating existing health disparities or as promoting health equity by providing marginalized groups and others greater opportunities to achieve better health.

This coalition could be sustained by imposing licensing fees to access the resources that would be imposed on commercial entities only. This coalition would also recognize that the creation and maintenance of standards involves “political and ethical work” (38) and thus could also be a learning community and community of practice that could provide a national forum for deliberation, debate, and ongoing development. The national coalition could also play a policy organizing and advocacy role. Specifically, this group could advocate for increased state and federal investment in upgrading health technology, specifically in under-resourced healthcare facilities to advance equitable access to health AI. This would build on government investments in health technology such as the Telehealth Network Grant program and The Health Information Technology for Economic and Clinical Health (HITECH) Act.

### Limitations

Because this is a qualitative research study, it is not broadly representative of the views of people who work at minority health organizations, or who work on health disparities and health equity in academic institutions, healthcare provider organizations, or in the private sector. In addition, participants were not recruited randomly, and the majority of our participants are from academic institutions, which is likely connected to the authors' own background as researchers at an academic institution. However, four of the six respondents from minority health organizations serve in roles that explicitly focus on advocacy. Although most of our respondents are academics, it is important to note that they are working on health disparity research which itself is marginalized in academia (39, 40). One of our respondents (who works at a health provider organization, but also conducts research) shared that that his work on health disparities is marginalized because of the difficulties he experiences trying to publish work on equity issues. In addition, though demographic information was not collected, during the interviews one-third of the participants identified themselves as members of the marginalized communities they serve (Respondents: 10002, 10010, 10011, 10012, 10015, 10017, 10018) and mentioned how their positionality and shared identity were early motivators for their health equity work.

Another limitation of this study is that it does not provide technical recommendations, such as how to build and test algorithms that advance health equity. However, it is our hope that our themes provide strategic priorities and directions for the development of health equity-centric health policy for AI, which can include technical developments. Our sample of participants is skewed towards academics, and those in the Southern and Western regions of the US, which is another limitation. The overrepresentation of academics results from the snowball recruitment sampling strategy, which relied on one author (KF) recruiting from her own professional network, which is largely comprised of academics. This over-representation of academics likely affects the key themes that emerged from our study. For example, since data is central to the work of most academics, the emphasis on connecting equitable AI health policy with data could reflect this academic bias. The theme focusing on bringing together multiple institutions and strategies may also reflect an academic bias, as universities often play a coordinating role, often through grant funding, between different institutions, such as healthcare centers, the surrounding community, and government and the private sector. The overrepresentation of academics in the Southern, more specifically Maryland and Virginia, may also present a skew towards more collaborative approaches with federal agencies because of pre-existing relationships between their respective institutions and certain policymakers, given their geographic proximity.

## Conclusion

Health AI represents an exciting area of technological development. The successes of health AI are numerous, but there are unfortunately already examples of how this emerging technology can harm those who already experience higher burdens of disparate and inequitable health care (41, 42). This study aimed to solicit the thoughts of those who have been working on health disparities for underserved groups. This is the first study to focus on the views of this specific group on the development of health AI policy for health equity. Other similar studies have focused on the perspectives of healthcare actors such as clinicians, patients, and researchers and have been largely concerned with the development and implementation of healthcare AI (29, 30). Research which has focused on healthcare AI policy has not explicitly focused on health equity or on the views of health equity professionals (43–45).

Results of this study showed that those working in health disparities and health equity bring important perspectives to the developing area of policy for health AI. Participants' responses reflected their commitment to showing the relationship between historical and structural issues and health treatment and outcomes. Their attention to data as a root cause of bias in health AI, to their discussion of power asymmetries and economic considerations are important contributions that can be incorporated into building more comprehensive policy for health care AI that can mitigate health disparities and open new pathways toward health equity.

## Data Availability

The datasets presented in this article are not readily available because due to the IRB protocol, informed consent forms, and data protection agreement, video and audio files cannot be shared publicly. Given the details shared in interviews and the limited number of health equity professionals in the United States, the research term found that the risk of re-identification is too high in de-identified transcripts. However, the interview guide may be shared upon request. Requests to access the datasets should be directed to the corresponding author or jhmeirb@jhmi.edu.
