# Supplementary material for: Perspectives on healthcare artificial intelligence policy from health equity professionals: findings from an interview study
Source: Front Digit Health. 2026 May 7;8:1797705. doi: 10.3389/fdgth.2026.1797705 (PMC13190201; doi:10.3389/fdgth.2026.1797705)
Supplement: Supplementary file 1 [file Datasheet1.pdf]

Supporting Information: Interview Guide

Advancing Health Equity in AI/ML in Health Regulation and Policy

Principal Investigator: Kadija Ferryman, PhD

Johns Hopkins University

Research Assistant: Odia Kane, PhD, MPH

Johns Hopkins University

<u>In-Depth Interview Guide</u>

This interview is going to explore how input from organizations that focus on racial and ethnic minority health and/or health disparities could inform policy development and FDA regulatory guidance for AI tools in medicine.

As a reminder, you can stop the interview at any time if any question makes you feel uncomfortable, or choose to skip any question you wish.

Background Information/Career

First, let’s start off with your background with [organization].

How long have you been on staff at [organization]?

Can you please describe what you do at [organization]?

Where is your organization most active?

Probe: In certain cities, states, regions?

Health Disparities & Health Equity

How do you/your organization define health disparities?

How do you/your organization define health equity?

Has your organization supported/endorsed any federal policies that target health disparities?

Probe: Has your organization written any letters of support, organized call/email campaigns, or mobilized constituents for a specific piece of legislation/regulation?

Do you think your organization has had an impact on health equities and/or health disparities?

Probe/Clarify: How does your organization define impact?

Why or why not has your organization had an impact on health disparities or health equity?

[If yes] Which activities/efforts have had the most impact? Why? Which have had the least? Why?

How do you think your organization has the most impact overall?

Have you worked with other organizations on advancing health equity?

[If not] Do you have plans to connect with other organizations or agencies?

AI in Health/Medicine

Some examples of artificial intelligence tools in medicine include the use of an AI software to analyze medical images, or the use of an AI model or software to predict risks of developing an illness. These are just a couple of examples. I'd like to ask you a few questions about AI in medicine.

Do you know of any specific examples of problems with the use of AI in medicine?

How does your organization define artificial intelligence?

Does your organization have a position or perspective on the use of AI tools in medicine?

Probe: What specific ideas or concerns do you think your organization's target demographic may have about AI tools in medicine?

AI in Medicine Policy

Does your organization have a position or perspective on the use of AI tools in medicine to affect health disparities or advance health equity?

Has your organization been involved in any policy work (with the FDA or other agencies) related to AI in medicine?

Could your organization become more involved in policy development for AI in health/medicine? Why or why not?

Who do you see as the key actors and institutions operating at the national level in AI in medicine policy development?

Probe: Do these actors or institutions represent communities of color?

Probe: Do they meaningfully engage and incorporate communities of color and center their interests in the policy process?

Probe: Are there organizations that do meaningful work that are not being included in these key institutions?

Has your organization collaborated with other actors to address this area?

Probe: Are you aware of any cross-org collaborations in this area**?**

Probe: How are people and communities of color positioned within policy networks?

Probe: Whose voices are most powerfully connected across and within networks?

Probe: Whose voices need more representation across and within networks?

Do you know of relevant events within which AI in health policy is being enacted and implemented?

Probe: How have these events affected or involved communities of color?

Probe: How salient or significant are the effects of events on communities of color?

Future of AI in Medicine

From your perspective, are there economic and/or political contexts that affect AI in medicine policy?

Probe: Are these economic and political contexts disproportionately affecting communities of color?

Probe: What role do communities of color have in shaping these contexts?

How do you think AI will be used in medicine in the near future? What about in the long term?

Do you think AI tools can mitigate health disparities now or in the future?

Probe: If not, why not?

Probe: If yes, how? Are there specific AI tools that are more effective or promising than others?

Probe, if necessary: In what ways may AI in health/medicine be limited when it comes to advancing health equity?

Do you have any suggestions for improvements "on the ground" regarding the use of how AI in medicine is currently being used?

Is there anything else you'd like to share related to AI, health disparities, and policy development? Or anything I/we have missed?
