# Supplementary material for: Perspectives on healthcare artificial intelligence policy from health equity professionals: findings from an interview study
Source: Front Digit Health. 2026 May 7;8:1797705. doi: 10.3389/fdgth.2026.1797705 (PMC13190201; doi:10.3389/fdgth.2026.1797705)
Supplement: Supplementary file 2 [file Datasheet2.pdf]

Supporting Information 2. Sample Codes and Consensus Discussion

Parent Code	Subcodes	Description
Definitions	Health disparity/disparities	Description of health disparities in response to the definition question in the interview guide
	Health equity	Description of health equity in response to the definition question in the interview guide
	Artificial Intelligence/Machine Learning	Description of AI/ML in response to the definition question in the interview guide
Health Equity/SDOH/Disparities	In reference to systems of oppression **and** the impact on health, healthcare, and health outcomes	
Systems of Oppression/Advantaged-Disadvantaged Groups	In reference to systems of oppression outside (though related to) healthcare	
Policy	**Include:** All forms of state/federal legislation and regulatory policies. And if the policy extends/affects more than employees of a particular institution<br><br>**Exclude:** organization specific internal policies	
Research	Description of research completed by agencies or institutions	

In our first codebook, the "Policy" code included any mention of a policy or policies. After coding the first transcript, the research team decided to focus the "Policy" code on legislation (local, state, or federal), regulation, and organizational policies that impact the community to exclude policies for organization employees (e.g. weekly check-ins). We also had an extensive discussion about combining or separating the "Health Equity" and "Systems of Oppression" codes. It was ultimately decided to keep the two codes separate because participants may speak about systems of oppression where health equity isn't mentioned (e.g. barriers to community engagement work) or speak about health equity without mention of systems of oppression (e.g. generally advocating for universal healthcare).
